# Execution of new trajectories toward a stable goal without a functional hippocampus

**DOI:** 10.1002/hipo.23497

**Published:** 2023-02-16

**Authors:** Adrian J. Duszkiewicz, Janine I. Rossato, Andrea Moreno, Tomonori Takeuchi, Miwako Yamasaki, Lisa Genzel, Patrick Spooner, Santiago Canals, Richard G. M. Morris

**Affiliations:** ^1^ Centre for Discovery Brain Sciences, Edinburgh Neuroscience University of Edinburgh Edinburgh UK; ^2^ Department of Psychology University of Stirling Stirling Scotland UK; ^3^ Department of Physiology Universidade Federal do Rio Grande do Norte Rio Grande do Norte Brazil; ^4^ Instituto de Neurociencias, CSIC‐UMH San Juan de Alicante Spain; ^5^ Department of Biomedicine, Danish Research Institute of Translational Neuroscience (DANDRITE) Aarhus University Aarhus C Denmark; ^6^ Department of Anatomy, Graduate School of Medicine Hokkaido University Sapporo Japan; ^7^ Donders Institute for Brain, Cognition, and Behaviour Radboud University and Radboudumc Nijmegen The Netherlands

**Keywords:** electrophysiology, hippocampus, pharmacology, spatial learning, spatial navigation

## Abstract

The hippocampus is a critical component of a mammalian spatial navigation system, with the firing sequences of hippocampal place cells during sleep or immobility constituting a “replay” of an animal's past trajectories. A novel spatial navigation task recently revealed that such “replay” sequences of place fields can also prospectively map onto imminent new paths to a goal that occupies a stable location during each session. It was hypothesized that such “prospective replay” sequences may play a *causal* role in goal‐directed navigation. In the present study, we query this putative causal role in finding only minimal effects of muscimol‐induced inactivation of the dorsal and intermediate hippocampus on the same spatial navigation task. The concentration of muscimol used demonstrably inhibited hippocampal cell firing in vivo and caused a severe deficit in a hippocampal‐dependent “episodic‐like” spatial memory task in a watermaze. These findings call into question whether “prospective replay” of an imminent and direct path is actually necessary for its execution in certain navigational tasks.

## 1 INTRODUCTION

Efficient memory‐guided navigation in mammals is thought to be mediated in diverse ways. Two prominent examples include (1) access to knowledge embedded in cognitive maps, which are allocentric mental representations of the spatial layout of an environment (O'Keefe & Nadel, 1978); and (2) path integration, which involves a continuous updating of current positional information based on self‐movement coupled to the computation of a return vector to any start point (McNaughton et al., 2006; Mittelstaedt & Mittelstaedt, 1980). The importance of the hippocampus as a biological substrate for a cognitive map was suggested by the discovery of place cells, and is supported by many causal lines of evidence including lesion, pharmacology and molecular genetic studies (McHugh et al., 1996; Morris et al., 1982, 1986; Tsien et al., 1996). Theoretical models of path integration (McNaughton et al., 2006) are supported by data showing that the hippocampus can sometimes be involved in this process (Whishaw, 1998). Other navigational strategies include learning an egocentric orientation from a start point and/or approach to specific landmarks.

Correlational physiological data has been extremely important in building a picture of spatial processing. Recordings of various types of spatially‐tuned neurons in the hippocampus and associated brain regions in freely‐moving rodents have revealed further cell types beyond place cells including head‐direction cells (Peyrache et al., 2019; Taube et al., 1990a, 1990b), grid cells (Fyhn et al., 2004; Hafting et al., 2005), border (Lever et al., 2009; Solstad et al., 2008) and object‐vector cells (Deshmukh & Knierim, 2013; Høydal et al., 2019). Both place cells and grid cells have been observed to fire in trajectory‐like sequences (Foster & Wilson, 2006, 2007; Lee & Wilson, 2002; O'Keefe & Recce, 1993; Ólafsdóttir et al., 2016; O'Neill et al., 2017; Pfeiffer & Foster, 2013; Skaggs et al., 1996; Wikenheiser & Redish, 2015). Off‐line hippocampal place cell sequences were first observed during non‐rapid eye movement sleep episodes after a period of running persistently along a linear track (Wilson & McNaughton, 1994). These offline sequences are present during short bursts of high frequency oscillations called sharp wave‐ripples (SWRs). They sometimes reflect prior experience and have also been implicated in memory consolidation (Buzsáki, 1989; Girardeau et al., 2009; Gridchyn et al., 2020), but see Dragoi and Tonegawa (2011).

Place cell sequences are, however, also present when the animal is awake and performing a navigation task. They can occur concomitantly with theta oscillations (Colgin, 2013; Dragoi & Buzsáki, 2006; Foster & Wilson, 2007; O'Keefe & Recce, 1993; Skaggs et al., 1996; Wikenheiser & Redish, 2015), or be compressed in time during awake SWRs (Foster & Wilson, 2006; Jadhav et al., 2012). In a landmark study by Pfeiffer and Foster (2013), rats were trained to shuttle, each day, between a novel daily “Home” location and multiple “Random” locations in a large arena. Importantly, a minority of SWR‐associated trajectory sequences were observed to contain information about future paths from Random to Home locations. Whether the Home locations were really encoded and then recalled in an explicit sense is unclear, but this finding, recently replicated in a similar task on a smaller but more complex arena (Widloski & Foster, 2022), raised the tantalizing possibility that the hippocampus may be involved in the active planning of spatial trajectories toward future goals.

These physiological observations are, however, *correlational* and observed in a task whose status with respect to involving allocentric spatial memory or involving path‐integration is unclear. Notwithstanding these differing representational aspects, the question arises of whether such neural activity in the hippocampus is on the neural pathway *causal* to the execution of such movement trajectories or, alternatively, whether spatial trajectories computed elsewhere are merely being reported to the hippocampus. To compare these two possibilities, we trained rats on the same spatial navigation task in which future trajectory sequences had been observed (Pfeiffer & Foster, 2013). We first established comparable performance to that observed by them and then inactivated the dorsal and intermediate hippocampus pharmacologically. The aim was to determine whether hippocampal activity during ongoing navigation is indispensable. Various control experiments were conducted in parallel, notably to establish a drug concentration sufficient to block cell firing in the dorsal and intermediate hippocampus of anesthetized rats in vivo and to disrupt performance in an ‘episodic‐like’ allocentric spatial memory task in a watermaze.

## 2 MATERIALS AND METHODS

### 2.1 Ethics and reproducibility statement

Growing interest in the replicability of biomedical studies has led us (and others) to be explicit about blinding and other procedures as advised by the CAMARADES consortium (CAMARADES, 2019). The behavioral studies were conducted by experimenters JIR and AJD who were “blind” to the drug infused in any animal. The main study was conducted in two separate cohorts to examine whether comparable data was obtained in each (it was, and the data was combined). The electrophysiological experiment could not be conducted “blind” by AM as the effects of muscimol were so dramatic, but it did include counterbalanced vehicle infusions and all monitoring and measurements were conducted automatically. The animals were handled carefully and all surgery conducted with suitable anesthetic and post‐operative management of any pain. All procedures were compliant with the UK Animals (Scientific Procedures) Act 1986 and with the European Communities Council Directive of 24 November 1986 (86/609/EEC) legislation governing the maintenance of laboratory animals and their use in scientific experiments.

### 2.2 Animals

The subjects were adult male Lister Hooded rats (200–300 g on arrival; *n* = 73; Charles River, UK) housed in groups of 3–4 for the duration of the study. The experimental cohorts were as follows:

**Table:** 

**Cohort 1**: Behavioral study of Home‐Random navigation task, *n* = 10 **Cohort 2**: Hippocampal drug infusions and acute electrophysiology, *n* = 17 **Cohort 3**: Hippocampal drug infusions in Home‐Random navigation task, and watermaze delayed matching‐to‐place task (after surgical implantation of bilateral hippocampal cannulae), *n* = 15 **Cohort 4**: Hippocampal or medial prefrontal cortex (mPFC) drug infusions in Home‐Random navigation task (after surgical implantation of bilateral hippocampal and mPFC cannulae), *n* = 13, two animals excluded from mPFC dataset due to off‐target implants **Cohort 5**: mPFC drug infusions in Home‐Random navigation task (after surgical implantation of bilateral mPFC cannulae), (*n* = 14) (Supporting information) **Cohort 6**: Muscimol diffusion in the mPFC and hippocampus (using fluorescent muscimol bodipy, *n* = 4; Supporting information)

The animals had access to food and water ad libitum and were kept on a 12‐h light: 12‐h dark schedule (lights on at 6 am; behavioral testing conducted during light phase). For the arena experiments, food was restricted (20–25 g per day) and the animals kept at between 85% and 90% of free‐feeding weight. The animals were handled for at least 3 days before the start of all behavioral procedures.

### 2.3 Behavioral apparatus and tasks: Home‐Random navigation task

Arena experiments were conducted in an apparatus based upon that used by Pfeiffer and Foster (2013). It consisted of a square open field (1.6 m × 1.6 m, made of Plexiglas) with transparent walls (0.3 m), surrounded by 2‐dimensional extramaze cues on the walls of the experimental room as well as 3‐dimensional cues suspended from the ceiling (Figure S1A). There were four start boxes, one on each side of the arena, with doors remotely controlled by the experimenter. The arena floor was made of a 7 × 7 grid of identical removable square panels (10 mm depth). Each panel included a central liquid feeding location, created by drilling with a 19 mm ball cutter to a depth of 6 mm, with a 4 mm hole drilled in the center for the liquid dispenser connection (internal diameter 2 mm). The holes were connected to liquid reward dispensers that were controlled manually by the experimenter through an extended set of tubes. They were shaped so that a central portion could dispense liquid reward (banana‐flavored milkshake, 0.5 mL) to be available to the animals on any visit, this liquid being fully contained below the floor level. An overhead camera, associated DVD recorder and software were used to monitor the paths taken by the animals and to measure both latencies to traverse between locations and associated path lengths. The behavioral procedures were modeled closely on the original report of Pfeiffer and Foster (2013).

#### 2.3.1 Habituation

Rats first underwent several sessions of shaping to drink liquid reward and arena habituation. This began with training to transverse a linear track (1.5 m long × 0.15 m wide) to obtain liquid reward at either end, with one 20 min session per day until they reached the criterion (20 laps in 20 min) for three consecutive sessions. There were then five sessions of habituation on the arena (one 25 min session per day). Rats started each session in one of the start boxes (chosen in a quasi‐random sequence across sessions) and were free to explore the arena when the experimenter remotely opened the start box door. During session 1 and 2, all wells were initially filled with liquid reward and refilled every 5 min. During sessions 3–5, one well per quadrant was initially filled with reward; it was re‐filled after the rat had consumed the previous reward, and then left that particular quadrant.

#### 2.3.2 Main task

Daily training on the navigation task itself consisted of continuous running between reward locations. For analysis purposes, this was divided into “trials”, each consisting of two “phases” called “Home” and “Random.” In the Home phase, the rat would start at a previous Random location and approach the Home location at which liquid reward would be delivered upon arrival. This daily Home location was not marked by any local cue but was one in which the location of reward availability was stable within a session but changed between sessions. In the immediately following Random phase, the animal would leave the Home location to search all over the arena for wherever reward would be delivered, one location having been silently filled with reward while the animal was at the Home location; a Random location was defined as being one of 25 varying locations available across trials on that session, these also being changed between sessions. These two phases completed each successive trial giving two measures of latency and path‐length (Random to Home, and Home to Random). Without interfering with the animal, the next trial began immediately, in continuous mode as in the original study of Pfeiffer and Foster (2013). Sessions began with a “Start Box to Random” search path, which was not included in the behavior analyses.

#### 2.3.3 Drug infusion sessions

Following the 8 training sessions, 2 counterbalanced drug infusion sessions (drug, vehicle) were interleaved with 1 regular training session. They involved quite large bilateral infusions of 2 μL of artificial cerebrospinal fluid (aCSF) or muscimol (1.3 mM) into the hippocampus or mPFC (prelimbic region). The drugs were given 40 min prior to the start of the session (see below).

#### 2.3.4 Focus of the analyses

The animals performed up to 25 trials per session (or 25 min total time on the arena). However, satiation differentially affected the animals on the latter trials of any session, leading us to focus our analysis on only the first 10 trials (20 rewards) of each session. There were 8 initial sessions of training in all experiments.

### 2.4 Behavioral apparatus and tasks: Watermaze task

The watermaze, with associated extramaze cues and overhead video monitoring equipment was used as previously described (Morris, 1984). Trial 1 (T1) of each session was given as a rewarded probe test, and this was done using an “on‐demand” or “Atlantis” platform (Burešová et al., 1985; Spooner et al., 1994).

#### 2.4.1 Procedure

The procedure of the “episodic‐like” delayed matching to place (DMP) protocol in the watermaze task is described in detail elsewhere (Rossato et al., 2018; Steele & Morris, 1999). In this task, the hidden escape platform moves from one location to another between sessions, directly analogous to the moving Home location in the Home‐Random arena navigation task. This version of the watermaze is hippocampal‐dependent, with effective learning of the new daily platform location requiring the integrity of sufficient tissue in the dorsal hippocampus (Hoz et al., 2005) and its functional integrity with respect to fast synaptic transmission, plasticity and dopaminergic neuromodulation (O'Carroll et al., 2006; Riedel et al., 1999; Steele & Morris, 1999). Performance is typically characterized by a long escape latency on T1 as the animal searches for the platform whose location on that session is still unknown, followed by rapid 1‐trial location learning that occurs during the 30 s period on the escape platform which rose to near the water surface after 60 sec swimming. The allocentric memory of where escape was possible on the last session is followed by relatively direct paths to that location on T2–T4 from any start location in the pool, with a small but significant residual memory last through to the next day's session.

#### 2.4.2 Main task

Four trials per session needed to be used, rather than the 25 trials of the Home‐Random navigation task, as asymptote is reached within 2–3 trials. The intervals between trials and escape platform locations were described in detail in Rossato et al. (2018). In this study, there were also 8 sessions of initial training.

#### 2.4.3 Drug‐infusion sessions

As in the Home‐Random navigation task, drug‐associated sessions followed the 8 training sessions and consisted of 2 counterbalanced drug infusion sessions interleaved with 1 regular training session. They involved bilateral intrahippocampal infusions of 2 μL of aCSF or muscimol (1.3 mM). The drugs were given 40 min prior to the start of the session.

#### 2.4.4 Focus of the analyses

The primary measure of performance computed was the time taken (in sec) until the animal first crossed the correct location (12 cm diameter) where the platform would become available (T1) or was available (T2–T4)—the “first crossing latency.” We also computed path length which, given stable swimming speeds, is directly correlated with latency. The secondary measure was, during T1 only, the time spent swimming in a virtual zone of 40 cm diameter centered on the location of the platform during the previous session (“24‐h memory retention”). This time is normalized and represented as a percentage relative to the 4% area of the pool that the zone occupies. This 4% level represents “chance” if the animal were to be swimming around the pool randomly. Having both measures provided a measure of daily learning within the domain of short‐term memory (as in the Home‐Random navigation task), and separately a measure of 24‐h memory retention.

### 2.5 Surgery

Guide cannulae for drug infusions were implanted into either the hippocampus or mPFC. Anesthesia was induced using isoflurane (induction, 5%, maintenance, 1%–2%; air‐flow, 1 L/min). The animals were then placed in the stereotactic frame (David Kopf Instruments, USA). Bilateral 26‐gauge steel guide cannulae were inserted (for the dorsal hippocampus, single cannulae, 4.4 mm length in each hippocampus; for the mPFC, dual cannulae, 4.9 mm length, 1.5 mm distance between cannulae; Plastics One, USA) with stylets (33 gauge) that protruded 0.5 mm below the end of the cannula. Cannula implantation coordinates were as follows: *hippocampus* (from bregma): anterior–posterior (AP), −3.96 mm; mediolateral (ML), ±3.00 mm; and dorsal‐ventral (DV) from the dura, −3.00 mm; *mPFC*: AP, +3.00 mm; ML, ±0.70 mm; and DV, −2.50 mm. When surgery was completed, the animals received a sub‐cutaneous injection of the analgesic rimadyl, were allowed to recover on a heating pad until normal behavior resumed, and then returned to the vivarium where they were closely monitored over the ensuing days.

### 2.6 Drug infusions

For drug infusions, the stylets in the guide cannulae were replaced with two single infusion cannulae (for the hippocampal infusions, 33 gauge; Plastics One), or with a double infusion cannula (for the mPFC infusions, 33 gauge; Plastics One) or connected to two 10 μL microsyringes (Hamilton, USA) in a microinfusion pump (Native Instruments, Germany) via flexible plastic tubing filled with Fluorinert (3 M, USA). The tips of infusion cannulae projected 0.5 mm below the intracerebral tip of the guide cannulae.

For all infusions, 2 μL of drug per cannula was infused at 0.5 μL/min; this is a relatively large volume which risked infusions spreading beyond the target structure, but it was essential to maximize the possibility of intra‐regional spread of effect. The infusion cannulas were left in place for a further 2 min to aid drug absorption before being replaced with stylets. The drug infusions were performed 40 min prior to the start of critical test sessions, this interval being based on data from electrophysiological recordings in vivo. The rats were habituated to the experimental procedure of injection for several days before the infusion sessions in order to minimize stress.

The concentration of muscimol (MW = 114.1; Tocris Bioscience, UK), a γ‐aminobutyric acid type A (GABA_A_) receptor agonist, used was 1.3 mM (2.6 nanomoles per site of infision; 0.16 μg/μL). We used aCSF at pH 7.2 (8.66 g/L NaCl, 224 mg/L KCl, 206 mg/L CaCl_2_·2H_2_O, 163 mg/L MgCl_2_·6H_2_O, 214 mg/L Na_2_HPO_4_·7H_2_O, 27 mg/L NaH_2_PO_4_·H_2_O) in H_2_O as a vehicle and for control infusions. Both vehicle and drug solutions were stored in 20–50 μL aliquots at −20°C until use.

### 2.7 In vivo hippocampal electrophysiology

The aim of the electrophysiology studies was to establish an effective location, dose and volume of muscimol that would successfully inhibit cell firing in the hippocampus in vivo.

#### 2.7.1 Apparatus

We used a stereotaxic apparatus (David Kopf Instruments) under non‐recovery urethane anesthesia (1.3 g/kg body weight), with the first intraperitoneal injection of urethane given to the animals during brief isoflurane anesthesia (4% isoflurane in 0.8 L/min O_2_). The stimulating electrode was a twisted bipolar Teflon‐coated platinum‐iridium electrode (20 μm diameter, 400 μm coated diameter for each of the two single strands) aimed at the angular bundle of the perforant path. The recording electrode in the majority of experiments was a single Teflon coated platinum‐iridium wire targeting the hilus of the dentate gyrus. In a subset of animals, the recording electrode was a DiI‐coated multichannel silicon probe of 32 channels with linear profile and 100 μm spacing between contacts (NeuroNexus) which spanned the whole dorso‐ventral profile of the hippocampus across 3.2 mm. The drug cannula was a 28‐gauge stainless steel tube whose tip was stereotaxically located at least 1 ± 0.4 mm away from the recording electrode. The recording electrode coordinates were: *dorsal hippocampus* [from bregma: AP, −3.00 mm; mediolateral (ML), ±1.80 mm; and dorsal‐ventral (DV) from the dura, −3.20 mm]; *intermediate hippocampus* (AP), +5.45 mm; ML, ±3.50 mm; and DV, −3.50 mm. When advancing the recording electrode, the electrophysiological signal was monitored for hallmark signs of crossing the CA1 and DG cell layers to ensure proper electrode placement within DG.

#### 2.7.2 Extracellular field potential recording

Conventional field potential recordings were made, with stimulation every 20 sec, and these monitored and calculated online using custom software (EPS software; PS). In response to biphasic 200 μs stimulus pulses of circa 600–800 μA, we measured both the early‐rising slope of the evoked potential by linear regression over several points, and the amplitude of the evoked population spike in the dentate gyrus. The stimulus intensity was adjusted to secure initial population spike amplitudes of circa 3–6 mV. Once acquired using suitable electrode placements, potentials typically remained relatively stable over periods of up to 3–4 h, with a small upward drift of the population spike [but not the field excitatory postsynaptic potential (fEPSP)] that rarely exceeded 15% over this long period. Animals for which the potentials were unstable were discarded. The same long time‐period stability was observed when aCSF was infused into the dorsal hippocampal formation at a depth targeting stratum lacunosum‐moleculare of the CA1 area. A volume of 2 μL at 1.3 mM was infused that, on the basis of previous autoradiographic and electrophysiological data (Morris et al., 1989; Rossato et al., 2018) has been shown to diffuse throughout the entire CA1, CA3 and dentate gyrus regions of the dorsal and intermediate hippocampus.

### 2.8 Histology and diffusion profile of fluorescently labeled muscimol

We conducted routine Nissl staining of brain sections from animals in the behavioral studies to determine the site at which the implanted cannulae were located in the hippocampus and mPFC. Animals with cannulae implanted off‐target (*n* = 2, mPFC implants) were removed from the analysis.

To identify and confirm visually the site of infusion and spread of maximal concentration of muscimol in mPFC and hippocampus, we infused fluorophore‐conjugated muscimol (FCM) from the stereotaxically defined sites of the guide cannulae. We hoped to use this drug to quantify the extent of diffusion. However, its much higher molecular weight (FCM, MW = 607.46; muscimol, MW = 114.1) and likely greater lipophilic properties proved problematic, but we report the results for transparency. FCM was dissolved in aCSF (1.3 mM; Hello Bio, UK). Cannulae positions and the drug infusion procedure were same as the behavioral experiment. The sites of infusion and spread of the drug were assessed 40 min after its bilateral infusion into the mPFC and the dorsal hippocampus. Brains were removed and shock‐frozen on powdered dry ice, and 50‐μm‐thick coronal sections were acquired with a cryostat (CM1900; Leica Biosystems, Germany), and mounted on Silane‐coated glass slides. Bright field and fluorescent images of serial sections equally spaced 100 μm were taken with a BZ‐X700 Microscope (Keyence, Japan). Fluorescent images in grayscale were shown as arbitrarily assigned color display mode (pseudocolor) according to their gray levels within a range of 0–90 (A.U.). Overlaid images of bright‐field and pseudocolor images were made using Photoshop (Adobe Systems, USA).

#### 2.8.1 Focus of analysis

For the histology, the location of the tips of the cannulae was marked on appropriate sections of the rat brain atlas. For FCM, each area of interest was equally divided into grid squares (200 × 200 μm) and the averaged fluorescent intensity was measured. Also, we divided the entire cortical area at the injection point (i.e., hippocampus: −3.96 mm from the Bregma) into 10 equal regions, calculated the averaged fluorescent intensity, and set the threshold as the mean plus two standard deviations. The measurements of fluorescent intensity and area was made using MetaMorph software (Molecular Devices, USA).

### 2.9 Data analysis and statistics

For all training cohorts except Cohort 1 in the Home‐Random navigation task, video recordings of all drug infusion sessions were re‐scored and rat movement tracked with custom software (PS) written in LabVIEW (National Instruments, USA). Rat paths were then processed and analyzed using custom software (AJD) written in Matlab (MathWorks, USA). Trajectories were smoothened with a box filter and small deviations in position (<2 cm) were interpreted as head movements and removed from the dataset. Additionally, time spent on reward consumption was removed from the analysis. Path analysis software was calibrated against manually tracked camera recordings to ensure that processed rat trajectories were accurate.

All data are expressed as mean ± S.E.M. Statistical analyses were performed using SPSS Statistics (version 24; IBM, USA). Statistical significance was always determined by ANOVAs or paired *t*‐tests with Bonferroni correction where appropriate. All statistical tests were two‐tailed.

## 3 RESULTS

### 3.1 Paths to a new stable home location are faster and more direct

**FIGURE 1:**
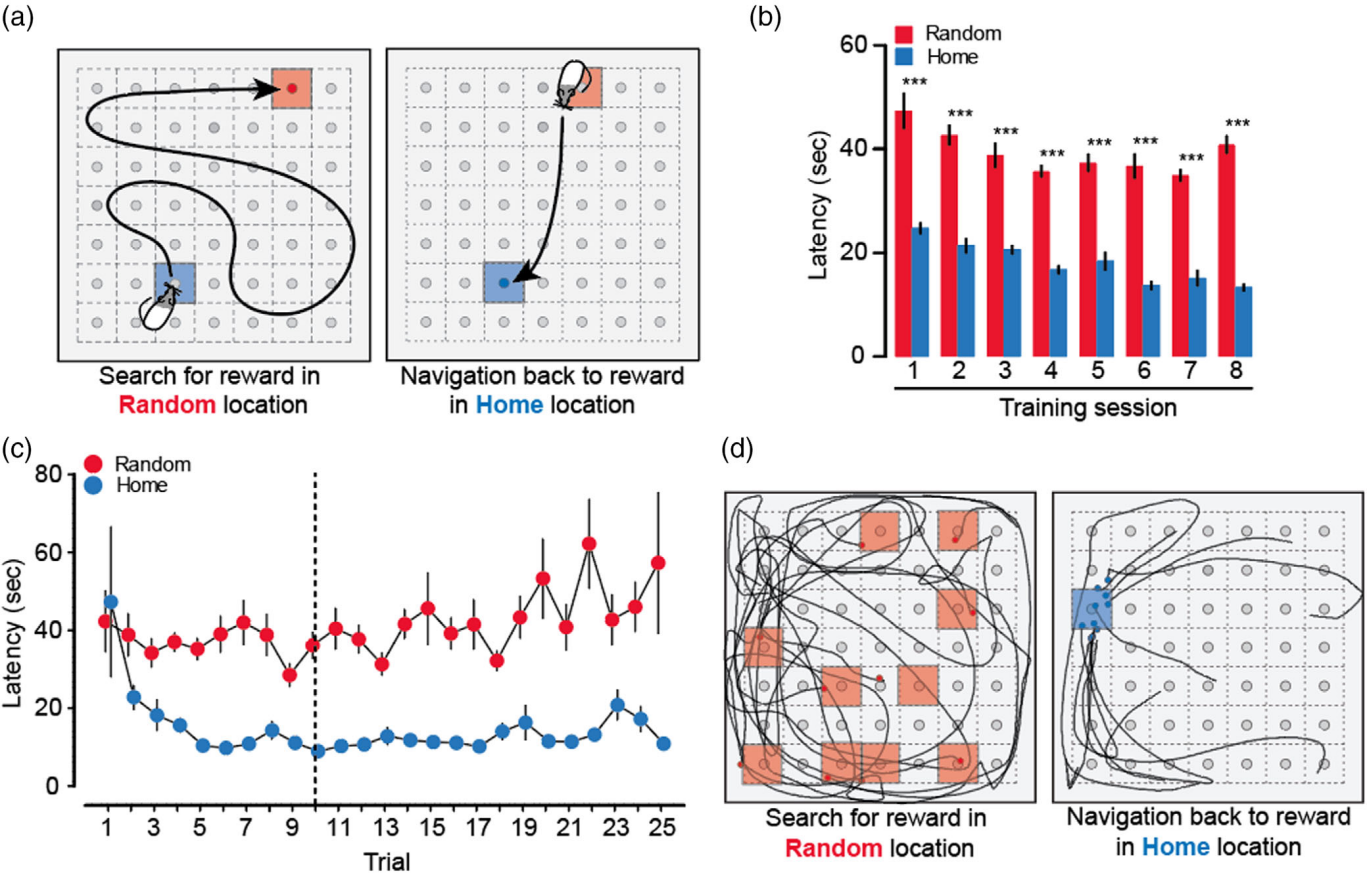
Behavioral analysis of the Home‐Random navigation task. (a) Experimental protocol. Rats of Cohort 1 alternated between search for reward in a Random location (red square) and navigation back to reward in the known Home location (blue square), which was kept constant within session but changed across daily sessions. (b) Task acquisition. Rats showed a robust difference in mean latency between Random and Home phases of the daily trials from the very first training session. (c) Within the 8th daily training session, latency to the rapidly learned new Home location decreased for the first few trials, reaching a stable plateau by Trials 4–10 (dashed line). In contrast, latency to the Random location varied from trial to trial. (d) Examples of paths to Random locations (left) and to Home location (right) from an individual training session. ***p < 0.001; see text for full statistical reporting; means ± S.E.M.

The first step was to establish that the task produced a behavioral pattern similar to that of Pfieffer and Foster (2013). In the Home‐Random navigation task, the rats of Cohort 1 (*n* = 10) learned to search all over the arena to find reward in a Random location (Random phase of a trial), and then return more quickly to the apparently rapidly learned Home location (Home phase), often with a relatively direct trajectory (Figure 1a; Figure S1A; see Section 2). A new Home location was introduced during each training session, and rats continuously performed the sequence of Random and Home phases within a trial for up to 25 trials.

From the outset of training, and across 8 sessions, the data revealed a robust difference in latency for the Home and Random phases of successive trials across all 8 training sessions (Figure 1b) (across sessions, *F*_1,7_ > 700, *p* < 0.001; pairwise comparisons for each session, *p* < 0.001). There was a steady improvement in performance across training sessions during both the Home (*F*_1,63_ = 20.7, *p* < 0.001) and Random phases (*F*_1,63_ = 5.71, *p* < 0.001) of successive sessions. By the final training session, this yielded average Home directed latencies of 10–12 s with minimal variation. The observed differences in performance during Home and Random phases are in agreement with those reported by Pfeiffer and Foster (2013).

Within each training session, journeys to the Home location took progressively less time over the course of circa 3–5 trials, reaching a successful asymptote by trials 4–10 (Figure 1c). In contrast, latencies during Random phases of successive training sessions did not decrease across trials within a daily training session and displayed greater variability as satiation developed on later trials (Figure 1c). On the final session of training (session 8), the animals retrieved the reward from the Home location three times faster than from a Random location—a difference also evident in the more direct paths taken to retrieve the reward from the Home location (Figure 1d). Further into the training session, rats started showing signs of declining motivation, and sometimes failed to complete all 25 trials within the time limit (note increased standard error in later Random trials in Figure 1c). These observations justified limiting the behavioral analysis of further cohorts to the first 10 trials of the session with our focus on whether the Home‐Random difference in performance was affected by hippocampal inactivation.

### 3.2 Muscimol blocks spiking activity in the hippocampus for several hours

**FIGURE 2:**
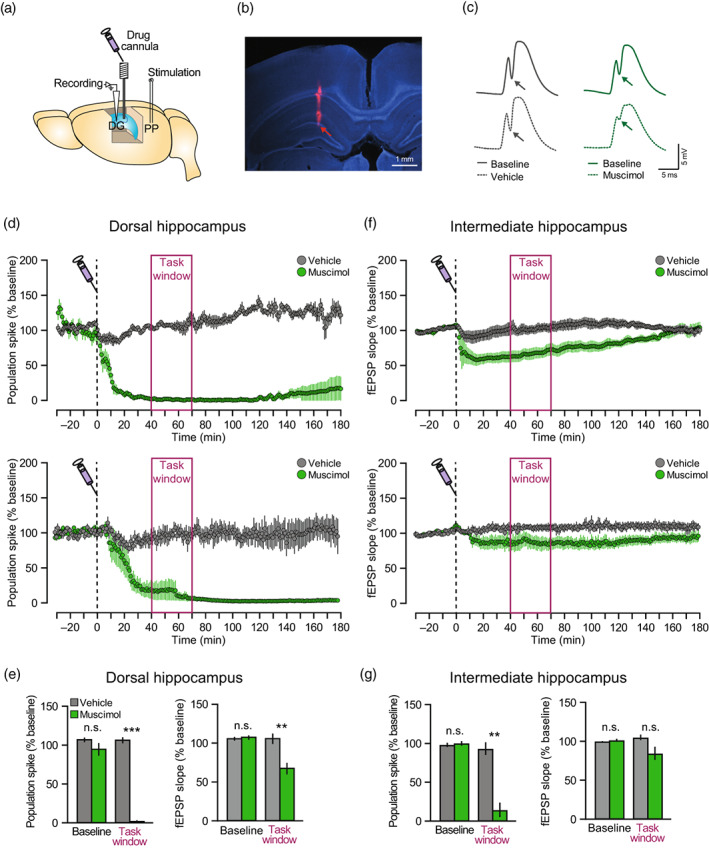
Electrophysiological assessment of the spread of muscimol in the hippocampus. (a) Schematic of the in vivo electrophysiological recording setup. Evoked activity induced by perforant path (PP) stimulation in dorsal and intermediate dentate gyrus (DG) was monitored in anaesthetized rats before, during and after infusion of muscimol into the dorsal hippocampus. (b) Representative image of a coronal section showing the tract of a DiI‐coated electrode (red) directed at DG (red arrow). (c) Representative evoked potentials recorded in DG before and after the infusion of vehicle or muscimol. Arrows indicate the downward slope of the population spike that is high reduced following muscimol infusion, and (d–g) Muscimol infusion into dorsal hippocampus of rats in Cohort 2 abolished the evoked population spike and interfered with synaptic transmission in (d, e) dorsal and (f, g) intermediate hippocampus. **p < 0.01; ***p < 0.001; n.s., not significant; means ± S.E.M.

The next step was to measure, using Cohort 2 (*n* = 17), the extent of hippocampal inactivation caused by local muscimol infusion which was calibrated across a range of doses (Figure 2a–c). The data presented is the optimum achieved, in which population spikes and evoked fEPSPs in the dentate gyrus of separate urethane‐anesthetized rats in vivo were recorded before, during and after infusion of the large volume of 2 μL of 1.3 mM muscimol or aCSF (Figure 2). This volume was chosen to maximize spread along the longitudinal axis of the entire hippocampus, including CA1, CA3, and DG. At this concentration, complete abolition of evoked population spikes in the dentate gyrus of the dorsal hippocampus (*n* = 5 rats in Vehicle condition, *n* = 4 rats in Muscimol condition) was observed during the time period of ‘Task‐window’ for the behavioral task (Figure 2d,e; Vehicle vs. Muscimol: *t*_7_ = 20.7, *p* < 0.001) together with a significant but incomplete decrease in slope of fEPSPs (Vehicle vs. Muscimol: *t*_7_ = 3.58, *p* < 0.01).

To examine longitudinal spread of effect, an additional set of recordings were performed in the intermediate hippocampus, 2.5–3.0 mm distant from the infusion site (*n* = 4 rats/condition, *n* = 8 total). The decrease in the population spike remained extensive along the longitudinal axis encompassing large parts of the intermediate hippocampus (Figure 2f,g; Vehicle vs. Muscimol: *t*_6_ = 5.52, *p* < 0.01), in contrast to a smaller and non‐significant effect on fEPSP slope (Vehicle vs. Muscimol: *t*_6_ = 1.93, *p* > 0.05).

The second measure of drug diffusion was conducted to quantify the anatomical spread of muscimol. Fluorophore‐conjugated muscimol (FCM, Allen et al., 2008; Bonnevie et al., 2013) was infused into the dorsal hippocampus in another cohort of rats (*n* = 4 hemispheres in two rats). It spread throughout anterior–posterior axis of the dorsal hippocampus (Figure S2A–C) with minimal spillover onto the overlaying dorsal and midline neocortex. Surprisingly, the spread across the hippocampal regions was somewhat more limited than that indicated by electrophysiological mapping. It is likely that the high molecular weight of FCM underestimates the spread of non‐conjugated muscimol.

Overall, dorsal hippocampal infusion of muscimol at this volume and concentration (see Section 2) is enough to abolish evoked spiking activity in the dorsal as well as intermediate hippocampus. Recordings in the ventral hippocampus were not conducted as this region is unlikely to be critically involved in spatial navigation, and single cell recordings of ventral hippocampal place cells were not made in that region in the original study of Pfeiffer and Foster (2013).

### 3.3 Hippocampal cell activity is not necessary for the execution of rapid new paths to a stable home location

**FIGURE 3:**
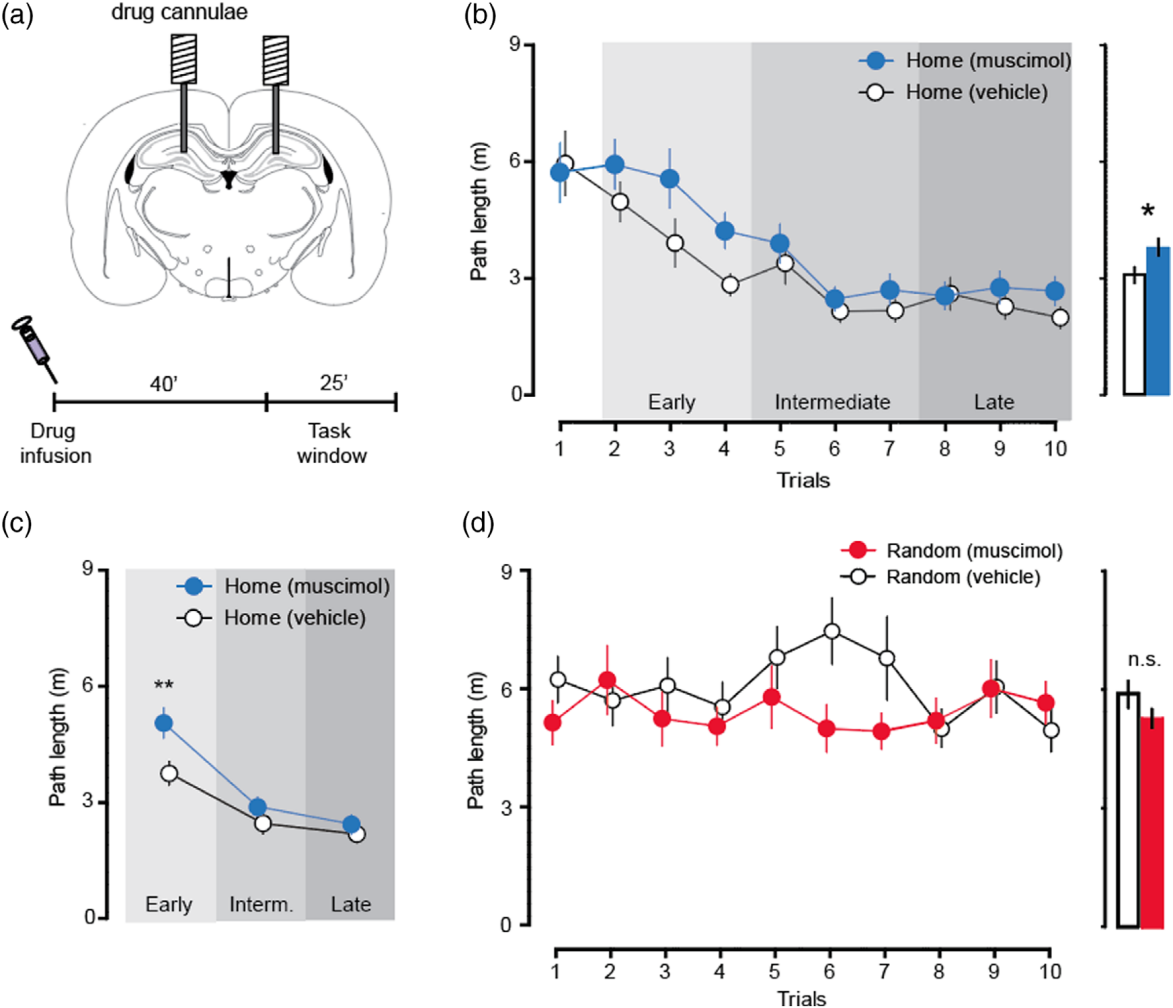
Hippocampal inactivation with muscimol did not substantially impair the Home‐Random navigation task. (a) Schematic of bilateral hippocampal la positions and timeline of the drug infusion and task‐window. (b) Hippocampal inactivation with muscimol in rats of Cohorts 2 and 3 did not substantially impair performance measured by path length in the Home‐Random navigation task. (c) Grouping of the data from panel B into early (T2–T4), intermediate (T5–T7) and late (T8–T10) trials of T2–T10 of drug infusion sessions, showed drug‐associated deficit only on T2–T4. (d) No impact of muscimol on path lengths shown across Random phases of the first 10 trials. *p < 0.05, **p < 0.01; n.s, not significant; means ± S.E.M.

Cohorts 3 and 4 (*n* = 28) were then used to assess the impact of blocking hippocampal cell activity using muscimol on navigation by the freely moving animals. After eight sessions of initial training, using animals implanted with bilateral drug cannulae directed at the dorsal hippocampus (Figure 3a; Figure S1B), the rats completed the drug and vehicle sessions in a counterbalanced manner. The electrophysiological data (Figure 2) indicate that neurons throughout the dorsal and intermediate hippocampus would not have been able to fire during the task‐window period of the behavioral study. Based on the original report, we reasoned that inactivating the hippocampus should impact on “prospective replay” and have a substantial deleterious effect on the ability of rats to navigate quickly from Random to Home locations across the daily trials.

Contrary to this prediction, we observed that rats were unaffected at asymptote—navigating just as fast to Home after muscimol infusion as after vehicle control (Figure 3b). However, in a direct comparison of muscimol and vehicle infusions in the Home phase, a modest muscimol‐induced impairment in the rate of decline of path length for Random‐to‐Home paths was observed (*F*_1,27_ = 5.04, *p* < 0.05; Figure 3b) although the comparison for latency was not quite significant (*F*_1,27_ = 3.42, *p* > 0.05); collectively, these data nonetheless suggest a modest slowing in the rate of learning to run directly to the Home location. To explore this apparent deficit further, we separated the successive analyzed block of 10 trials into three blocks of 3 trials [early (Trials 2–4), intermediate (T5–T7) and late (T8–T10) of each daily session, omitting T1 for which the animals could not yet know where Home was located; Figure 3c]. The observed impairment in path length was limited to T2–T4 when the Home location in each session was still relatively new (*F*_1,27_ = 7.42, *p* < 0.05; pairwise comparisons with Bonferroni correction: early, *t*_27_ = 3.58, *p* < 0.01; middle, *t*_27_ = 1.14, *p* > 0.05; late, *t*_27_ = 1.12, *p* > 0.05). Thus, in the later trials starting after T4, there was no muscimol‐associated impairment. Hippocampal inactivation did not affect the ability of rats to find the reward on the Random phase of a trial (path length, *F*_1,27_ = 2.87, *p* > 0.05, Figure 3d; latency, *F*_1,27_ = 0.01, *p* > 0.05). A more detailed presentation of these data is in Figure S3.

### 3.4 Muscimol blocks performance of an episodic‐like spatial memory task in the watermaze

While the electrophysiological data indicates that spiking activity would have ceased in the dorsal and intermediate hippocampus during task performance, we found that it had only a transient effect on the Home‐Random navigation task. Faced with this almost “null” result, we turned to a different behavioral task that is definitively hippocampal‐dependent—the “episodic‐like” delayed matching‐to‐place (DMP) task in the watermaze. This task requires animals to remember the most recent daily location of the escape platform which also changes from day to day; it is, however, a task which is learned in a definitively allocentric manner. A massive and highly significant deleterious impact of muscimol was observed in this task.

**FIGURE 4:**
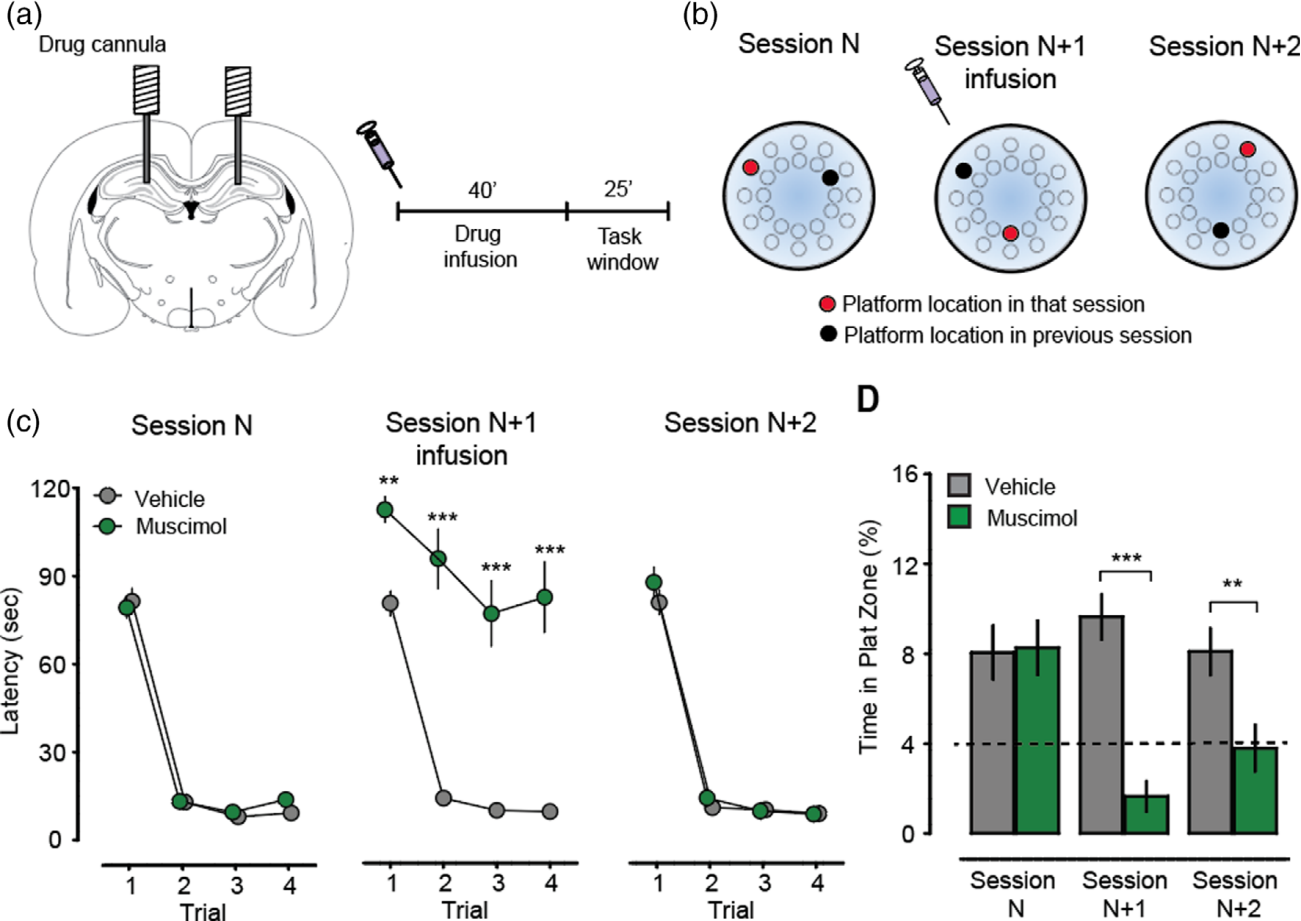
Hippocampal inactivation with muscimol did impair an episodic‐like spatial recency task in the watermaze. (a) Schematic of bilateral hippocampal cannula positions and timeline of the drug infusion and task window. The animals in Cohort 3 were used. (b) DMP protocol in the watermaze. The platform location was changed each session but fixed within the four daily training trials. The rats were assessed on their ability to recall the location of the platform on the previous session (black platform symbol; the first 60 s of T1) and encode the current session's location (red platform symbol; after 60 s on T1 and during T2–T4). (c) Muscimol impaired learning of the DMP task. (left) Session N showed striking reduction in escape latency between T1 and T2 of each session. Session N + 1: Muscimol infusion into dorsal hippocampus caused a recall impairment and a severe impairment in the ability to learn the new platform location. Session N + 2: Learning returned to normal. (d) Muscimol infusion impaired recall of the previous session's platform location both on the session of infusion (Session N + 1) and the following session (Session N + 2), without affecting new learning on session N + 2. The impairment on Session N + 2 in the absence of muscimol was due to muscimol having been present on Session N + 1 to block encoding of the Session N + 1's platform location. **p < 0.01, **p < 0.001; means ± S.E.M.

Using again the same animals of Cohort 3 (*n* = 15), the animals learned to search for the varying location of the hidden escape platform each session during an initial set of 5 sessions (Figure 4a). Thereafter, repeated “blocks” of 3 successive sessions (Session *N*, *N* + 1, *N* + 2; with muscimol only given on the *N* + 1 sessions; Figure 4b) were given as a counterbalanced within‐subjects design to examine the impact of intrahippocampal muscimol or vehicle injections (Figure 4a). Using the Atlantis platform on T1 of each session with the escape platform unavailable until 60 s had elapsed, relatively random searching for the new daily platform location was accompanied with a small but highly significant search tendency toward the platform location of the previous session. There was some day‐to‐day memory, but it was modest. On T2–T4 of each session, the escape platform was always available (within 1.5 cm of the water surface). Muscimol caused a devastating effect during Session *N* + 1. While first‐crossing latencies for the new platform location in the vehicle‐treated animals decreased strikingly from T1 to T4, they stayed at the same level in the muscimol‐treated animals (Trial × Drug interaction, *F*_3,120_ = 4.63, *p* < 0.01). For individual trials, latencies in muscomol‐treated animals were significantly higher on T2–T4 (T2: *t*_14_ = 8.03, T3: *t*_14_ = 6.59, T4: *t*_14_ = 7.18; *p* < 0.001 for all comparisons), with a small increase compared to vehicle‐treated animals on T1 (*t*_14_ = 3.13, *p* < 0.01).

The DMP task also offers the opportunity of examining whether a goal location learned on Session N is remembered on the first trial of Session *N* + 1—an important test of allocentric coding as the start locations in the watermaze vary from session‐to‐session as well as from trial‐to‐trial. The escape platform unavailable for the first 60 s of T1 using the Atlantis Platform, this period of the 120 s Trial 1 serving as a “probe” of spatial recall and enabling the zone‐analysis of search focus. In the absence of any drug or vehicle infusions (Session *N*), search time in the target zone occupying 4% of the surface area of the pool on Session *N*–1 was approximately twice the level that would be expected by chance (~8% time in platform zone, comparison to chance: Vehicle, *t*_15_ = 3.39, *p* < 0.01; Muscimol, *t*_15_ = 3.62, *p* < 0.01; comparison of drug conditions: *t*_15_ = 0.23, *p* > 0.05; Figure 4d, left). Animals treated with muscimol on Session *N* + 1 did not display this overnight memory (Figure 4d, middle) whereas vehicle‐treated animals continued to do so (comparison of drug conditions: *t*_15_ = 5.64, *p* < 0.001). After the muscimol had diffused away and washed out from the hippocampus by Session *N* + 2, muscimol‐treated animals still performed poorly on T1 of Session *N* + 2, reflecting their inability to learn the location trained on Session *N* + 1 (Figure 4d, right; comparison of drug conditions: *t*_15_ = 3.35, *p* < 0.01), but they then learned normally on T2–T4 (Figure 4c). Overall, these results demonstrate that the same pharmacological intervention used in the Home‐Random task impairs allocentrically encoded recency memory with respect to both learning within the day and recall the next day.

### 3.5 Lack of effect of medial prefrontal cortex (mPFC) inactivation on arena task

**FIGURE 5:**
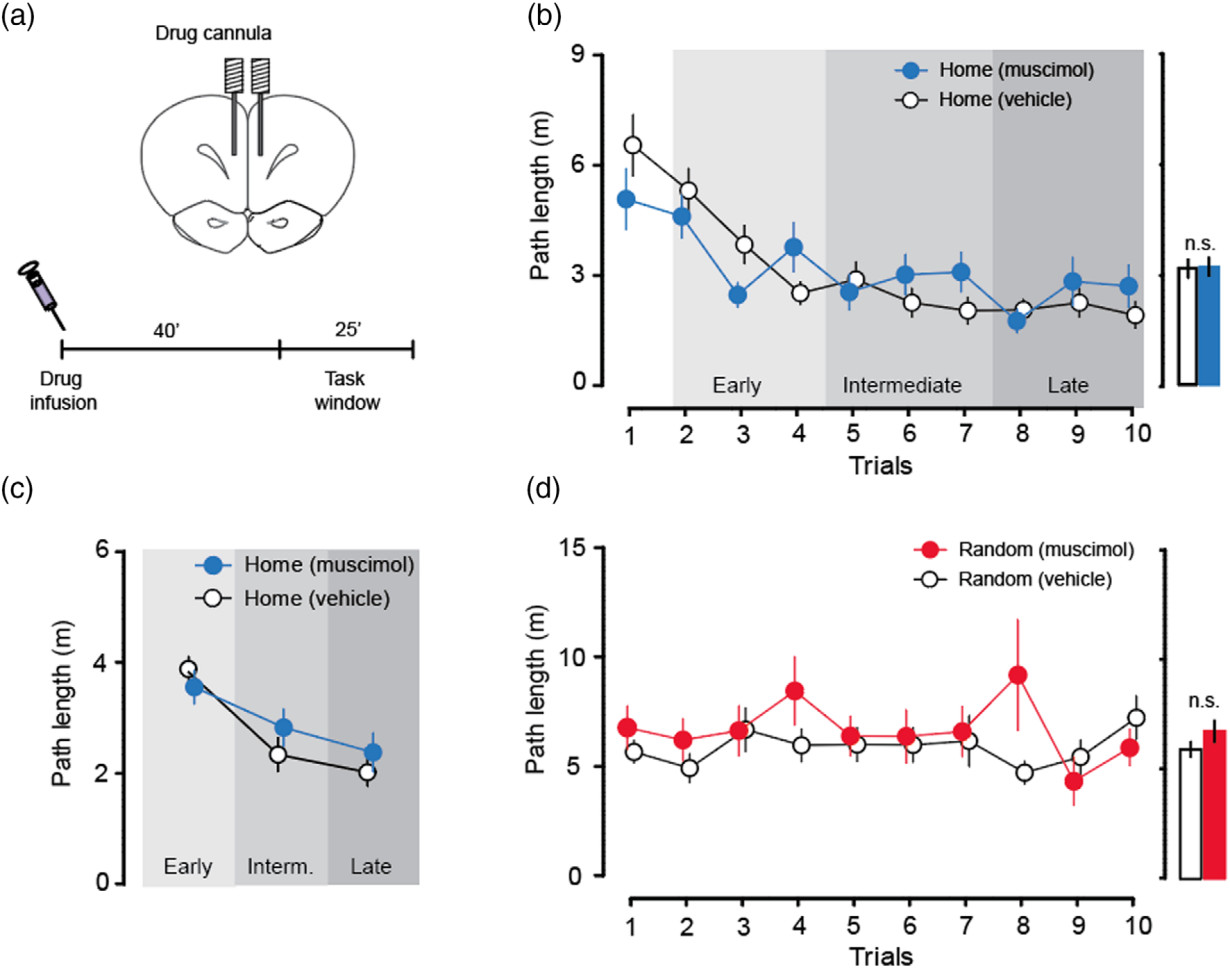
Inactivation of mPFC with muscimol did not affect the ability of rats to navigate to the Home location. (a) Schematic of bilateral mPFC cannula positions and timeline of the drug infusion and task window. (b and c) mPFC inactivation with muscimol had no effect on the path lengths to the home location. (d) mPFC inactivation had no effect on the path lengths in search of the Random location. n.s, not significant; means ± S.E.M.

Since the successive trials in the Home‐Random task are continuous, that is, occur without any experimenter interference or imposed delay, we hypothesized that, except for the early trials, the task does not engage long‐term memory mechanisms and the performance may instead be supported by working memory. Human and animal studies established that the prefrontal cortex is an important node in the working memory circuitry (Curtis & D'Esposito, 2003; Funahashi, 2017). We thus examined the impact of muscimol in the prelimbic region of mPFC, the prefrontal cortical area involved in working memory in rodents (Baeg et al., 2003; Bolkan et al., 2017; Spellman et al., 2015; Yang et al., 2014). We first trained two cohorts of rats [Cohorts 4 and 5, *n* = 25; data pooled for analysis] and implanted them with bilateral cannulae directed at mPFC (Figure 5a). Analysis of separate animals established that our muscimol infusions were on‐target (Figures S4A–C and 4 infusions in two rats). As in the case of hippocampal inactivation, intra‐mPFC muscimol caused no impairment in path length for Home‐to‐Random paths across trials (*F*_1,24_ = 1.75, *p* > 0.05; Figure 5d). Importantly, in contrast to hippocampal inactivation, Random‐to‐Home trajectories were unaffected by muscimol infusion into the mPFC (path length, *F*_1,24_ = 0.61, *p* > 0.05; latency, *F*_1,24_ = 3.91, *p* > 0.05; Figure 5b), even if the trials were separated into early (T2–T4), intermediate (T5–T7) and late (T8–T10) (pairwise comparisons with Bonferroni correction: early, *t*_24_ = 0.75; middle, *t*_24_ = 1.06; late, *t*_24_ = 0.81, *p* > 0.05 for all comparisons; Figure 5c). In the absence of either a working or long‐term memory deficit following muscimol in the Home‐Random task, it would seem that some other navigational process supports performance.

## 4 DISCUSSION

There are two main findings of this study. The *first* is that the successful inhibition of hippocampal cell‐firing has relatively minimal effect on the Home‐Random navigation task reported by Pfeiffer and Foster (2013). Specifically, despite bilateral hippocampal infusion of muscimol, we observed similar acquisition of the short latency/short path‐length asymptote during Home phases of successive trials as they had observed, and the same consistently longer latencies in the Random phases. The early trial adoption of this pattern was, however, slightly but significantly affected by muscimol compared to aCSF vehicle treated animals. The dose and volume of intra‐hippocampal muscimol was titrated to yield an inhibition of cell‐firing for >2 h, measured electrophysiologically in both dorsal and intermediate hippocampus, with the behavioral tests conducted during a 30 min task‐window of minimal cell‐firing. The *second* main finding is that this same dose of muscimol had a devastating effect on an allocentrically encoded delayed matching‐to‐place watermaze task (recency memory) which was used used as a positive behavioral control for the effectiveness of intra‐hippocampal muscimol microinjection. The performance of muscimol‐treated rats in the DMP task showed very little improvement over successive daily trials, closely mimicking the effect of complete hippocampal lesions in the same task (Bast et al., 2009).

What is the implication of this dissociation? We first discuss features of the experimental design that support these claims about the results, together with reference to additional findings, and then the key issue of the putative *causal* role that prospective replay events might be playing in this type of navigational behavior. That prospective replay might be part of a causal path is explicit in the abstract of the Pfeiffer and Foster (2013) paper where they write: “[these sequences are] … supporting a goal‐directed, trajectory‐finding mechanism, which identifies important places and relevant behavioral paths, at specific times when memory retrieval is required.” The heart of our interpretation is to suggest a critical difference between a true allocentrically encoded episodic‐like memory task and one involving path integration.

### 4.1 Sufficient inhibition of spiking activity by muscimol, and its regional diffusion in hippocampus

It is unlikely that insufficient muscimol was infused to disrupt hippocampal spiking activity in the awake freely moving rat during the Home‐Random navigation task. Local muscimol infusion into the dorsal hippocampus in anesthetized rats largely abolished evoked spiking activity in dorsal (99% decrease) as well as in intermediate hippocampus (85% decrease). We did not perform single‐cell recording of place cells in freely moving rats because published unit recording data is available showing the deleterious impact of muscimol (Bonnevie et al., 2013). For example, even with a lower volume and concentration (0.5 mg/mL, 0.3 μL; 1.5 nanomoles compared to our use of 2.6 nanomoles), there was a complete loss of place cell firing in the dorsal hippocampus adjacent to the infusion as well as a loss of grid‐like tuning by grid cells in the medial entorhinal cortex (MEC) (Bonnevie et al., 2013). The concentration used in our study was 2.5 times higher with a volume 7 times larger (in nanomolar terms, circa 17 times higher).

Bonnevie et al. (2013) also mapped the spread of muscimol (FCM) revealing a spread largely limited to the hippocampus and the cannula track. Our FCM mapping data also revealed infusion into the targeted site, with the drug remaining largely in hippocampus (and, in separate animals, in mPFC). However, the spread of the high molecular weight FCM underestimates the spread of the normal “non‐conjugated” muscimol used in the main experiments. The evidence for this claim is the robust blockade of evoked cell‐spiking activity observed in the intermediate hippocampus where FCM fluorescence was not detected. The functional impact, rather than mere diffusion, of an antagonist of AMPA (α‐amino‐3‐hydroxy‐5‐methyl‐4‐isoxazole propionate)/kainate‐type of glutamate receptors has also been studied using 2‐deoxyglucose mapping to reveal widespread hypometabolism (23% reduction) throughout the dorsal and intermediate hippocampus (Riedel et al., 1999). Deleterious effects on watermaze learning and memory consolidation were observed in this latter study. However, in that case, while the site of intrahippocampal infusion was comparable (and extending to the intermediate hippocampus), the rate of infusion of 0.5 μL/h for 24 h continuously over 7 days infusion was very different, achieved via chronically implanted osmotic minipumps. While one could argue that this chronic method of diffusion undermines the value of any comparison, the point is that there was a clear statistical association between the impairment of a behavioral task (watermaze) and a reduction of glucose utilization throughout the dorsal and intermediate hippocampus.

While we have not investigated whether muscimol infusions successfully inactivated the ventral‐most part of the hippocampus, we deem it unlikely that ventral hippocampus is capable of supporting navigation to the learned Home location to a higher degree than its dorsal counterpart. Ventral hippocampus has classically been associated with processing emotional, affective and/or contextual information (Bannerman et al., 2003; Fanselow & Dong, 2010). Its contribution to spatial navigation tasks is a matter of dispute (Bast et al., 2009; Fanselow & Dong, 2010; McDonald et al., 2018; Moser et al., 1995; Ruediger et al., 2012). Ventral hippocampal place cells have larger, less defined place fields (Jung et al., 1994; Royer et al., 2010) and show less theta rhythmicity (Royer et al., 2010) than their dorsal counterparts, which makes them less suited for accurate representation of spatial trajectories but perhaps sufficient to achieve context discrimination. Moreover, in awake rats, sharp‐wave ripples (SWRs) in ventral hippocampus occur independently of those in dorsal hippocampus and are functionally coupled to separate less spatially selective downstream targets (Sosa et al., 2020).

Taken together, the lack of an effect on the Pfeiffer and Foster (2013) task despite near complete inhibition of hippocampal pyramidal cell firing indicates that the prospective replay in cell firing of precise spatial trajectories observed by them is unlikely to be directly mediating navigation in their open field, and the unaffected ventral hippocampus would not have been able to contribute to the same degree as a normal functional hippocampus.

### 4.2 Superficial similarity of the Home‐Random navigation and DMP watermaze tasks

In the Pfeiffer and Foster (2013) task, the animals shuttle continuously between Home and Random locations, with the fixed Home location only changed across sessions. In the DMP watermaze task, the animal swims from any of four separate start positions to a hidden escape platform whose location is also changed across sessions. On the face of it, the tasks have a conceptual similarity and both appear to involve spatial recency memory.

We shall argue, however, that this apparent conceptual similarity is only superficial. Numerous lines of data indicate that watermaze spatial learning is allocentric and involves some “explicit” memory of a location in space identified with reference to extramaze cues. The animals climb onto the platform and look around to see where they are. Importantly, they do not swim back to the starting point of any trial (which changes across trials). Even in situations such as the present watermaze training protocol where the wider extramaze context of learning had been learned prior to any drug infusions, the encoding of that session's location of safety within this allocentric framework is known to involve the hippocampus and associated structures (Steele & Morris, 1999). Using the DMP task, we observed both a complete failure to learn the new session's escape location in the presence of muscimol or to recall the previous session's location the next day in its absence. From this, we infer that the dose was enough to prevent both new spatial memory *encoding* in the presence of the drug and subsequent spatial memory *recall*. In terms of the longitudinal axis of the hippocampus, the DMP task is exquisitely sensitive to both lesions and AMPA receptor blockade restricted to the dorsal and intermediate hippocampus (Hoz et al., 2005; Riedel et al., 1999), and the effect of muscimol in our DMP experiment was essentially the same as the effect of complete hippocampal lesions and much more severe than the effect of partial lesions (Bast et al., 2009).

Still, it is of note that we did observe a modest impairment in the initial learning of the new Home location in the early trials of the Home‐Random task. The animals with an inactive hippocampus required a few extra trials to match the asymptotic performance of the control group. This is consistent with the observed failure to remember the new platform location in the DMP task on subsequent trials of the same day. The hippocampus thus does play some role in the initial rapid learning of the new goal location, but this function seems to be orthogonal to the subsequent ability to navigate back to the goal once its location has been encoded.

### 4.3 The Home‐Random navigation task is likely a path integration task

Pfeiffer and Foster's (2013) beautiful data show unambiguously that prospective replay is playing out a representation of future trajectories in a brain structure implicated in spatial navigation. Their supposition was that this matters causally for accurate spatial navigation from Random to Home. But is successful navigation in their Home‐Random navigation task really an allocentric task that is dependent on the hippocampus? Our data suggest not.

Understanding the difference between the Home‐Random navigation task and the DMP watermaze task is the critical issue here, but first a word about what “hippocampal‐dependence” means. This term in widespread use was first defined in terms of the sensitivity of a task to lesions, but other interventions such as drugs, regional‐specific genetic manipulations, and both optogenetic and chemogenetic procedures are displacing the older lesion criteria in new ways. For example, some drugs such as N‐methyl‐D‐aspartate (NMDA) receptor antagonists impair spatial memory *encoding* without effect on *retrieval* (Steele & Morris, 1999). This is still a hippocampal‐dependent profile, but different from that obtained with lesions. Very low concentrations of muscimol can, under some circumstances, impair retrieval without any effect on encoding, a finding consistent with dendritic computation (Rossato et al., 2018), but such concentrations are an order of magnitude lower than those used here for which muscimol impairs both new learning and episodic‐like recall. Other interventions such as intrahippocampal blockade of AMPA receptors impair both encoding and retrieval of other allocentric spatial tasks (Riedel et al., 1999). Interestingly, the supposition that path integration is “hippocampal‐dependent” was made primarily on the basis of the impact of lesions of the fornix (Maaswinkel et al., 1999; Whishaw, 1998; Whishaw et al., 2001), but it is not supported by hippocampal lesion studies (Alyan & McNaughton, 1999). Hippocampal involvement in path integration has not, to our knowledge, yet been investigated in animals using more contemporary approaches that intervene directly with hippocampal physiology, but study of patients with definitive hippocampal damage has revealed no impairment of path integration (Shrager et al., 2008).

We doubt the Home‐Random navigation task, at least in its plateau phase, is either episodic or allocentric, whereas the DMP watermaze task is an episodic‐like task as usually defined (Clayton et al., 2003). This is because the animal remembers an event (escaping the water—*what*) at a specific location (*where*) in a familiar allocentric framework and, specifically, the most recent occasion that this happened (*when*). As discussed in detail by Steele and Morris (1999), recall over trials T2–T4 involves remembering where this “event” happened most recently, followed by a natural tendency to head to the place where it happened (because navigation to that location had consistently brought safety). Disrupting hippocampal cell firing with muscimol should, as shown here, be devastating for such recency‐recall within the session. A modest but significant memory of the most recent learned location lasts 24 h (in vehicle treated controls), can be measured and is also impaired in rats treated with muscimol on the previous day. Another facet of the DMP watermaze task is that trials start and stop and have experimenter involvement in carrying the animals, whereas the successive phases and trials of the Home‐Random navigation task are continuous. This discontinuous versus continuous issue is relevant to path integration, and likely also relevant to a new navigational honeycomb maze task in which trials are discontinuous but the animal must, in successive phases of choice opportunity, approach a learned goal appropriately (Wood et al., 2018). We predict that the honeycomb task also would be severely impaired by intrahippocampal muscimol injection.

In the Home‐Random navigation task, in contrast, finding liquid reward at a specific location in this continuous task likely does not constitute an event in quite the same way. This assertion may seem self‐serving for our interpretation, but bear in mind that with up to 25 trials per session, the reward will be found in every session in one place 25 times *and* in numerous other places one or more times each. In short, reward is potentially available almost everywhere. Such a training protocol is ideally suited to path integration solution (McNaughton et al., 2006). The idea is that a brain system keeps track of and accumulates the animal's translational and rotational movements during the Random phase of each trial, and then uses the accumulated vectors to compute a direct path back to the starting position (in this case, the Home location). The accumulator is then reset. Importantly, in such a system, the animal *never* learns or needs to learn where the starting position is located in an allocentric sense. It may know it on the basis of other aspects of experience, but accurate paths do not require explicit spatial knowledge. With respect to the Home‐Random navigation task, the accumulator would have operated primarily during the Random search phases, with the return vector applied to get back to the start point of the random‐search. There is a longstanding debate about whether such path integration involves the hippocampal formation (McNaughton et al., 1996), with some data suggesting that it does (Maaswinkel et al., 1999; Whishaw, 1998; Whishaw et al., 2001) and others implicating instead the MEC (Banino et al., 2018; Burak & Fiete, 2009; Campbell et al., 2018; Tennant et al., 2018), the retrosplenial cortex (Elduayen & Save, 2014; Mao et al., 2020) and/or the head‐direction system (Butler et al., 2017; Valerio & Taube, 2012). Our muscimol infusions did not spread to the MEC, but similar muscimol infusions into the dorsal hippocampus have previously been shown to disrupt the periodic firing of grid cells (Bonnevie et al., 2013), indicating that entorhinal cortex is unlikely to support the Home‐Random differences in latency and path‐length (see also Shrager et al., 2008). The lack of effect of mPFC inactivation observed in this study indicates that prefrontal areas are also unlikely to serve as an anatomical substrate for path integration. Nevertheless, a path integration system, wherever located anatomically, might report the prospective trajectory to the hippocampus and this could be the basis of the SWR‐associated prospective replay sequences representing future trajectories. In our view, there are two separate points: there need be no causal link between these place cell sequences recorded in the hippocampus and the executed trajectories to the goal; and there is also no need for explicit allocentrically coded declarative memory of the Home location in the Home‐Random task. It is worth noting, however, that the recent modification of the Home‐Random task that includes intramaze barriers (Widloski & Foster, 2022) necessitates adoption of indirect trajectories to the goal location and may be more dependent on the SWR‐associated sequences. Establishing whether the addition of barriers makes the Home‐Random task hippocampal‐dependent is of future interest.

An alternative approach to explain the animal performance in the task is via Pavlovian place conditioning. That one location in the arena has reward multiple times and others only once per session, could nonetheless have served to increase the reward value of a particular area of the arena, with this location varying across sessions. The animals switch repeatedly between (a) directed approach to the Home location where they get reward multiple times (high‐value), and (b) a search strategy to diverse Random locations (low‐value), where they also get reward but only once for each location. The learning of value would then take place gradually using place conditioning. On this account, and in addition to path integration, the gradual increase in relative value of the Home location over 3–5 trials at the start of each session might have helped develop home path directionality, but not critically. Interestingly, a temporal‐difference conditioning algorithm for mediating directed approach to a valued location was developed by Foster et al. (2000) that does not appeal to declarative event memory but may nonetheless be relevant.

When we began this series of experiments, we had in mind to explore precisely timed optogenetic inactivation of the SWRs in the hippocampus that constituted the prospective replay. The notion was that halorhodopsin or ArchT disruption of the prospective path from Random to Home as represented by place‐cell sequences would reduce or even block the Home‐Random difference in latency and path length. The analysis just presented reveals that such a study is misconceived and, to the contrary, our analysis leads us to make the *opposite* prediction. Were it possible to direct exquisitely timed optogenetic inactivation throughout the Home to Random phase of a trial to a sufficient volume of the relevant brain structure(s) performing the path integration computations (wherever these are anatomically), only then would the faster latency to Home compared to Random, and lower path length, be reduced or abolished. In contrast, optogenetic disruption of SWRs at the start of the Home phases should be without effect. Such an experiment is, unfortunately, beyond the scope of our study.

### 4.4 The place of prospective replay in goal‐directed navigation

The analysis presented suggests, ironically, that animals in the Home‐Random navigation task never learn explicitly where Home is located each session, they merely execute paths computed using path integration to get them there accurately, aided modestly by some Pavlovian place conditioning of a non‐declarative nature. It is, therefore, worth pausing to reflect on Figure 1d; the differential pattern of the paths is striking; the radical claim being made here is that the animals need not to remember the Home location in any explicit sense. Newly learned goal locations can, nonetheless, be incorporated into the brain's cognitive map on multiple levels, including enriched representations of the goal in hippocampal place cell codes (Dupret et al., 2010; Hok et al., 2007; Hollup et al., 2001; Markus et al., 1995) and entorhinal grid cell codes (Boccara et al., 2019; Butler et al., 2019). This updating of the cognitive map with a new goal location, likely taking place in the early Random‐Home trials in the present study, could perhaps be aided by plasticity‐promoting neuromodulatory inputs to the hippocampus (Duszkiewicz et al., 2019; McNamara et al., 2014; Takeuchi et al., 2014, 2016). In bats, hipocampal cells can also encode distance and direction toward the goal (Sarel et al., 2017). The presence of the animal at a goal location is associated with awake SWR‐associated hippocampal replay events that include (Davidson et al., 2009; Diba & Buzsáki, 2007; Foster & Wilson, 2006; Pfeiffer & Foster, 2013), but are not limited to, representations of immediate past and future trajectories (Gupta et al., 2010; Karlsson & Frank, 2009). These findings highlight the possible importance of awake replay in ongoing navigation to and from goal locations. Interestingly, in a spatial memory task involving both a working memory component encoded in one trial and a long‐term spatial reference memory component encoded over many trials, awake SWRs depict trajectories associated with both components (Karlsson & Frank, 2009), but their disruption (Jadhav et al., 2012) or prolongation (Fernández‐Ruiz et al., 2019), respectively, impaired or enhanced only the working memory component, indicating that not all functions of awake SWRs are causally related to ongoing long‐term spatial memory.

The veracity of Pfeiffer and Foster's (2013) exciting results remains unchallenged by our findings. We did not monitor the single cell activity of hippocampal neurons during our investigation of the Home‐Random navigation task, and we recognize the importance of such prospective replay as indicating the likely output of a path integration system located outside the hippocampus. However, we did explore the dataset of Bonnevie et al. (2013) only to find that the electrode placement used for the purposes of their study was inappropriate for examining SWRs. An intact and functioning hippocampus gets to know and may even “need to know,” but we question the likely *causal* role of such reported sequences in the hippocampus in planning subsequent navigation. We are therefore obliged to end with an important qualification. Such a report to the hippocampus may not be essential for accurate navigation in this path integration task, but it may be very important for true allocentric tasks involving explicit memory (Jadhav et al., 2012; Kim & Frank, 2009) whereby it may query and/or consolidate the cognitive map via replay of a repertoire of possible trajectories within present and past environments (Gupta et al., 2010; Karlsson & Frank, 2009). To test these hypotheses, concomitant high‐density place cell recording or calcium imaging to identify and possibly disrupt specific prospective replay sequences during a definitively allocentric task would constitute a valuable follow‐up to the present work.

## AUTHOR CONTRIBUTIONS

*Conceptualization*: Richard G. M. Morris. *Methodology*: Adrian J. Duszkiewicz, Janine I. Rossato, Miwako Yamasaki, Tomonori Takeuchi, Andrea Moreno, Lisa Genzel. *Software*: Patrick Spooner. *Validation*: Janine I. Rossato. *Formal analysis*: Richard G. M. Morris, Adrian J. Duszkiewicz, Janine I. Rossato, Andrea Moreno, Santiago Canals, Miwako Yamasaki, Tomonori Takeuchi. *Investigation*: Richard G. M. Morris, Adrian J. Duszkiewicz. *Resources*: Patrick Spooner. *Data curation*: Adrian J. Duszkiewicz, Janine I. Rossato, Andrea Moreno. *Writing—original draft preparation*: Richard G. M. Morris, Adrian J. Duszkiewicz. *Writing—review & editing*: Richard G. M. Morris, Adrian J. Duszkiewicz, Tomonori Takeuchi. *Visualization*: Adrian J. Duszkiewicz, Tomonori Takeuchi. *Supervision*: Richard G. M. Morris, Santiago Canals. *Project administration*: Richard G. M. Morris, Santiago Canals. *Funding acquisition*: Richard G. M. Morris, Adrian J. Duszkiewicz, Tomonori Takeuchi. All authors discussed the manuscript.

## CONFLICT OF INTEREST STATEMENT

The authors declare that no competing interests exist.

## Supporting information


**FIGURE S1.** Behavioral apparatus and histology. (A) The open arena for the Home‐Random navigation task with wall‐placed and hanging 3D extramaze cues, and four start boxes (black) on each perimeter wall. (B and C) Histological identification of the cannula placement in the dorsal hippocampus (B) and mPFC (C). The numbers above each picture represent the distance (in mm) of the section from bregma (Paxinos & Watson, 2007) THE RAT BRAIN: In stereotaxic coordinates, 6th Ed, Netherlands, Elsevier.

**FIGURE S2.** Histological assessment of the spread of fluorophore‐conjugated muscimol (FCM) in the hippocampus. (A–C) Schematic of bilateral hippocampal cannula positions (A), representative images (B) and average fluorescence distribution of FCM (C) in the hippocampus (HPC) as well as two representative areas of the dorsal neocortex: parietal association cortex (PtA) and retrosplenial cortex (RSC). Means ± S.E.M.

**FIGURE S3.** Hippocampal inactivation with muscimol did not prevent the animals to successfully navigate to the known home location. (A–D) Latency (A and B) and path length (C and D) to goal during Random and Home phases after vehicle (A and C) and muscimol infusions (B and D) into the hippocampus. Means ± S.E.M.

**FIGURE S4.** Histological assessment of the spread of FCM in mPFC. (A–C) Schematic of bilateral mPFC cannula positions (A) representative images (B) and average fluorescence distribution of FCM (C) in the prelimbic region of mPFC (PrL) as well as two representative areas of the frontal cortex: secondary motor cortex (M2) and frontal cortex area 3 (Fr3). Means ± S.E.M.

## Data Availability

All data are available upon request.
