# Supplementary figures and images for: Execution of new trajectories toward a stable goal without a functional hippocampus
Source: Hippocampus. 2023 Feb 16;33(6):769–86. doi: 10.1002/hipo.23497 (PMC10946713; doi:10.1002/hipo.23497)

**Figure S1**

**A**

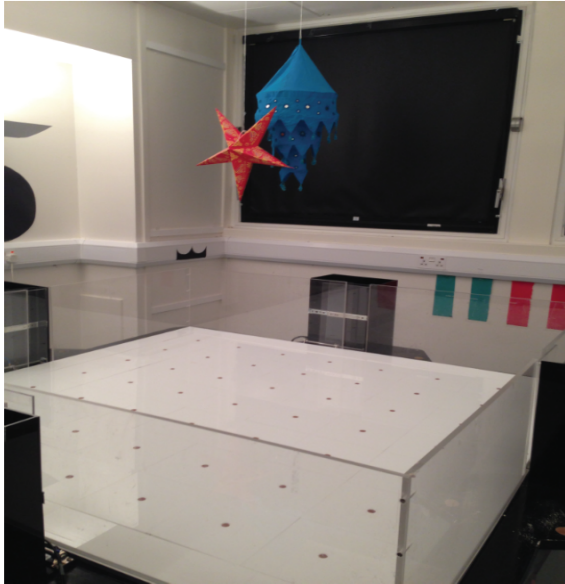

**B**

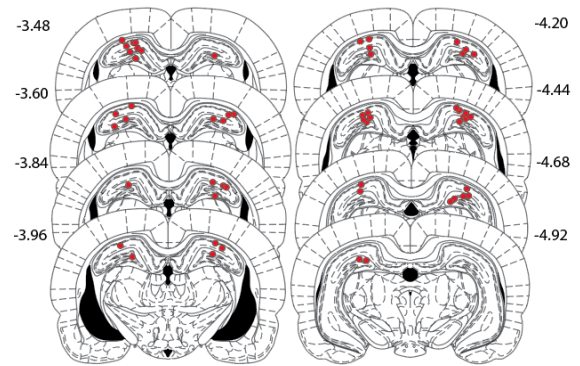

**C**

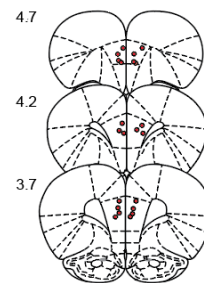

Supplement: Supplementary file 1 — FIGURE S1. Behavioral apparatus and histology. (A) The open arena for the Home‐Random navigation task with wall‐placed and hanging 3D extramaze cues, and four start boxes (black) on each perimeter wall. (B and C) Histological identification of the cannula placement in the dorsal hippocampus (B) and mPFC (C). The numbers above each picture represent the distance (in mm) of the section from bregma (Paxinos & Watson, 2007) THE RAT BRAIN: In stereotaxic coordinates, 6th Ed, Netherlands, Elsevier. [file HIPO-33-769-s003.pdf]

Figure S2

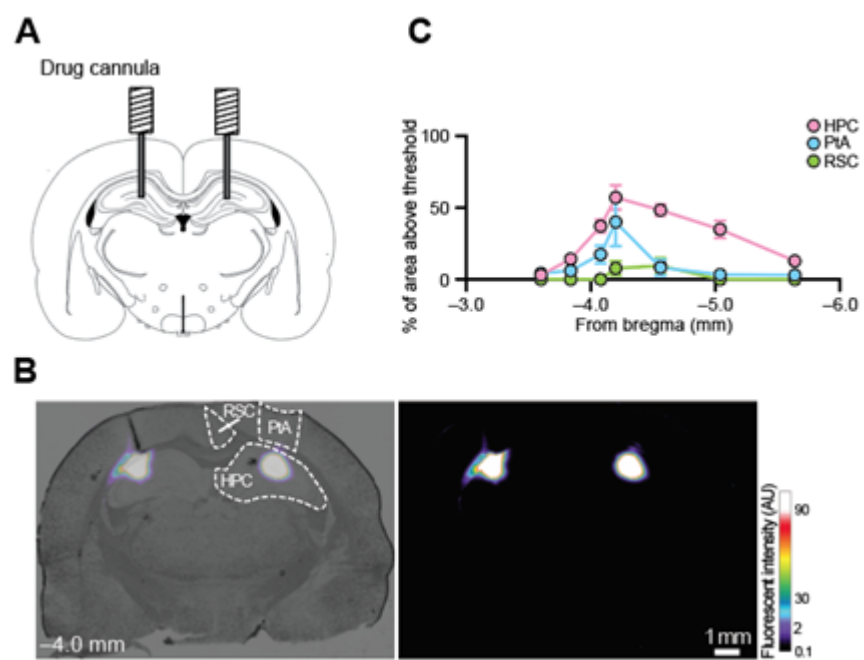

Supplement: Supplementary file 2 — FIGURE S2. Histological assessment of the spread of fluorophore‐conjugated muscimol (FCM) in the hippocampus. (A–C) Schematic of bilateral hippocampal cannula positions (A), representative images (B) and average fluorescence distribution of FCM (C) in the hippocampus (HPC) as well as two representative areas of the dorsal neocortex: parietal association cortex (PtA) and retrosplenial cortex (RSC). Means ± S.E.M. [file HIPO-33-769-s004.pdf]

Figure S3

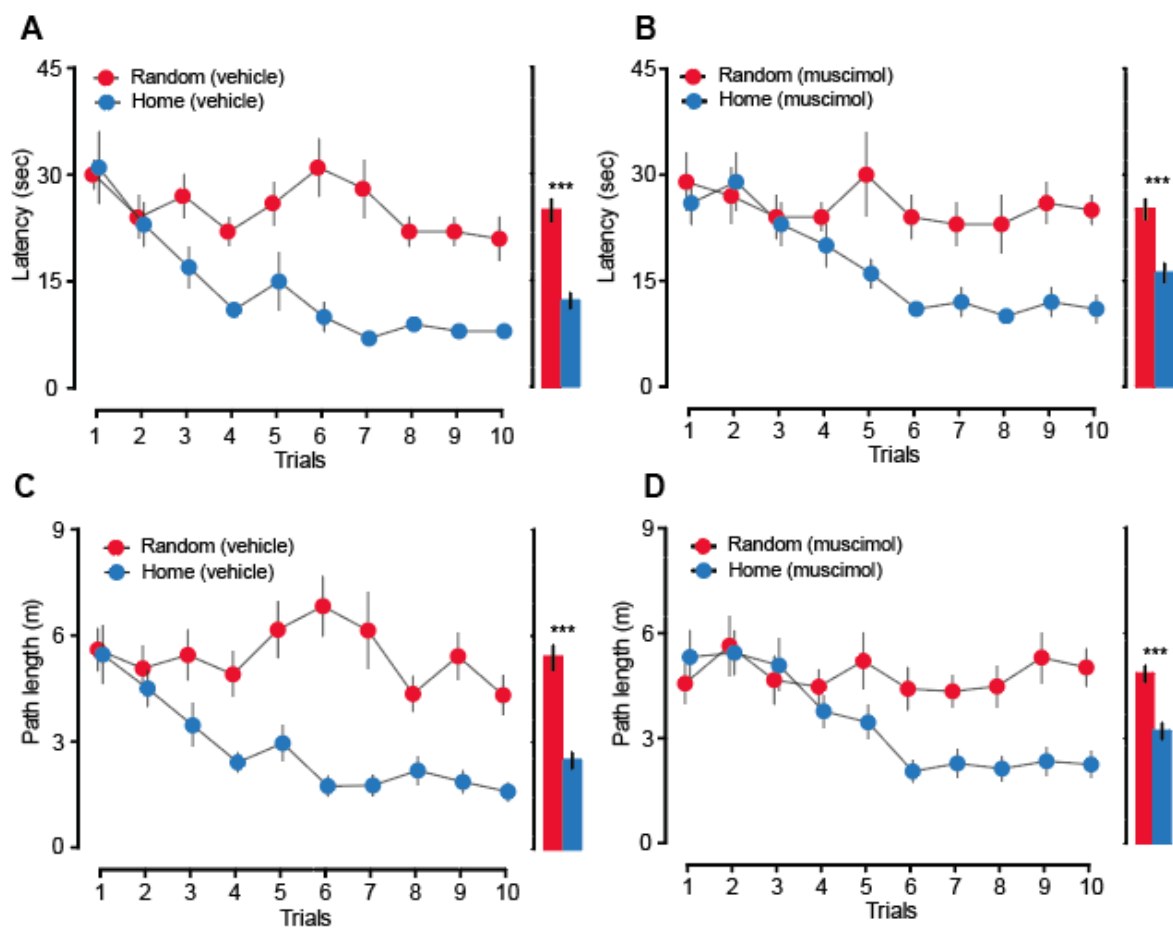

Supplement: Supplementary file 3 — FIGURE S3. Hippocampal inactivation with muscimol did not prevent the animals to successfully navigate to the known home location. (A–D) Latency (A and B) and path length (C and D) to goal during Random and Home phases after vehicle (A and C) and muscimol infusions (B and D) into the hippocampus. Means ± S.E.M. [file HIPO-33-769-s002.pdf]

Figure S4

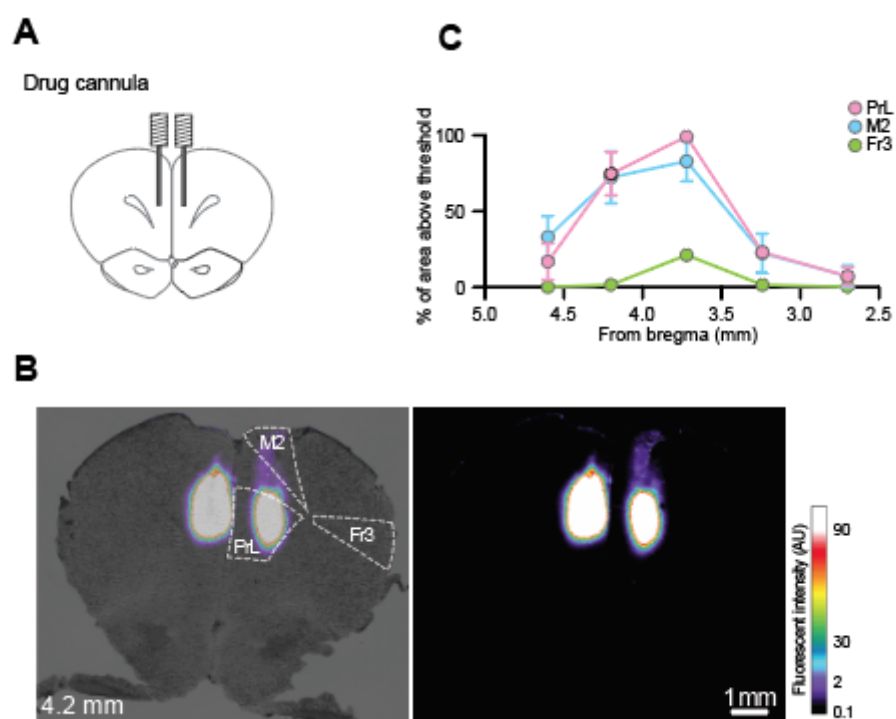

Supplement: Supplementary file 4 — FIGURE S4. Histological assessment of the spread of FCM in mPFC. (A–C) Schematic of bilateral mPFC cannula positions (A) representative images (B) and average fluorescence distribution of FCM (C) in the prelimbic region of mPFC (PrL) as well as two representative areas of the frontal cortex: secondary motor cortex (M2) and frontal cortex area 3 (Fr3). Means ± S.E.M. [file HIPO-33-769-s001.pdf]
